# Donepezil and Rivastigmine: Pharmacokinetic Profile and Brain-targeting After Intramuscular Administration in Rats

**DOI:** 10.22037/ijpr.2019.1100723

**Published:** 2020

**Authors:** Jana Zdarova Karasova, Martina Hrabinova, Marketa Krejciova, Daniel Jun, Kamil Kuca

**Affiliations:** a *Department of Toxicology and Military Pharmacy, Faculty of Military Health Sciences, University of Defence, Hradec Kralove, Czech Republic. *; b *Biomedical Research Centre, University Hospital, Hradec Kralove, Czech Republic.*; c *Department of Chemistry, Faculty of Science, University of Hradec Kralove, Hradec Kralove, Czech Republic. *

**Keywords:** Donepezil, Rivastigmine, Acetylcholinesterase inhibitors, Alzheimer’s disease, HPLC

## Abstract

Current palliative pharmacotherapy of Alzheimer’s disease based on the cholinergic hypothesis led to the development of four cholinesterase inhibitors. These compounds can bring prolongation of the symptom-free period in some patients. This is the first report directly comparing donepezil and rivastigmine plasma and brain levels in *in-vivo* study. Donepezil and rivastigmine were applied i.m. to rats; the dose was calculated from clinical recommendations. The samples were analysed on an Agilent 1260 Series LC with UV/VIS detector. An analytical column (Waters Spherisorb S5 W (250 mm × 4.6 i.d.; 5 μm particle size)) with guard column (Waters Spherisorb S5 W (30 mm × 4.6 mm i.d.)) was used. The mobile phase contained acetonitrile and 50 mM sodium dihydrogen phosphate (17:83; v/v); pH 3.1. The LLOQ in rat plasma was 0.5 ng/mL for donepezil and 0.8 ng/mL for rivastigmine, and the LLOQ in rat brain was 1.0 ng/mL for donepezil and 1.1 ng/mL for rivastigmine. Both compounds showed ability to target the central nervous system, with brain concentrations exceeding those in plasma. Maximum brain concentration after i.m. administration was reached in the 36 (8.34 ± 0.34 ng/mL) and 17 minute (6.18 ± 0.40 ng/mL), respectively for donepezil and rivastigmine. The differences in brain profile can be most easily expressed by plasma/brain AUC_total_ ratios: donepezil ratio in the brain was nine-times higher than in plasma and rivastigmine ratio was less than two-times higher than in plasma.

## Introduction

Alzheimer´s disease (AD) is a common age-related neurodegenerative dementia that has recently shown an alarming rise in the global population. In recent decades there has been considerable effort by many clinical and pre-clinical teams to understand the ethiology and pathophysiology of AD. The understanding of the disease origin and pathological cascade can help to find effective protection or a successful therapeutic strategy (1). Investigation has not produced any simple solution; the evidence indicates that AD is a complex disorder. The main hallmarks in AD pathogenesis are loss of cholinergic neurons, amyloid-β (Aβ) deposits in neuronal plaques, and neurofibrillary tangles (abnormally phosphorylated tau protein) (2, 3). Besides these main hallmarks, various other factors such as inflammation, oxidative stress, protein misfolding, metal ion accumulation, gene mutation, and neurotransmitter imbalance have been included in the AD pathological cascade. Despite these efforts, development of new target strategies has proved extremely difficult and most clinical trials have not yielded significant success (4). 

Current pharmacotherapy is based on the cholinergic hypothesis (2). This hypothesis explains the cognitive dysfunction as the result of loss of cholinergic neurons; the persistent deficit of acetylcholine is responsible for insufficient cholinergic neurotransmission (5). This led to the development of four cholinesterase inhibitors: tacrine (approved in 1993), donepezil (1996), rivastigmine (2000), and galantamine (2004). These compounds significantly improve the cognitive function and overall health of patients, but they only provide palliative therapy, prolonging the symptom-free period.

Donepezil is a commonly used relatively low-toxicity anti-AD drug that has shown beneficial effects on cognitive function, daily living activities, and some neuropsychiatric symptoms in AD patients (6, 7). Donepezil may also play some roles in mitigation of oxidative stress and improvement of cerebral blood flow (8, 9). 

Donepezil is classified as a short-acting or reversible agent since binding to acetylcholinesterase (AChE) is hydrolysed within minutes. Rivastigmine is intermediate-acting or pseudo-irreversible inhibitor of both cholinesterases with long-lasting AChE inhibition (up to 10 h) (10). The inhibition of both cholinesterases may provide additional benefits. In particular, the presence of high levels of butyrylcholinesterase (BChE) in brain structures such as the hippocampus, amygdala, and thalamus suggest that inhibition of both cholinesterases may have an important role in AD treatment (11, 12).

**Figure 1:**
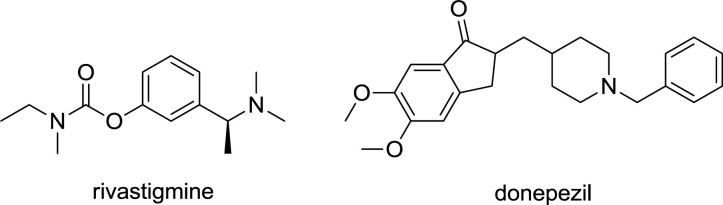
Structures of donepezil and rivastigmine

The aim of this study was to define the pharmacokinetics profiles of donepezil and rivastigmine after a single dose in rats (Figure 1). The dosages of both drugs were derived from doses recommended in human use (7). The doses that were finally calculated and applied (donepezil: 143 µg/kg and rivastigmine: 137 µg/kg) may be considered as relatively dose-equivalent. Pharmacokinetics studies already exist, describing brain-targeting of donepezil, but comparable/similar information about rivastigmine is still rare. Preclinical and clinical data for rivastigmine are limited to cholinesterase inhibition. Pharmacokinetics data based on pharmacodynamic interaction may not be accurate. According to our knowledge, this is the first report directly comparing the plasma and brain levels of donepezil and rivastigmine.

## Experimental

*Chemicals*

Donepezil and rivastigmine were purchased from Sigma Aldrich (Prague branch, Czech Republic). Chemicals for sample preparation and HPLC measurement were purchased from Sigma Aldrich (Prague branch, Czech Republic) and were of the best available quality: acetonitrile (ACN) and methanol in analytical grade; n-hexane and 1-butanol in gradient grade. All other reagents used in analytical methods and sample preparation were of analytical grade. The water used in the study was double-distilled and deionised HPLC-MS grade.

The internal standard solutions (IS; donepezil and rivastigmine) were prepared by solution in methanol to make final concentrations of 0.1 mg/mL (5 mg/50 mL). Stock solutions of the IS were stored at -20 ºC. A working IS being prepared daily from the stock solutions by dilution with 0.1% acetic acid (13).

*HPLC instrumentation*

The samples were analyzed on an Agilent 1260 Series liquid chromatograph (Palo Alto, CA, USA) composed of a degasser, quaternary pump, light-tight autosampler unit set, thermostated column compartment and a UV/VIS detector. Agilent ChemStation software (Palo Alto, CA, USA) and the statistical software Prism4 (Graph Pad Software, USA) were used for data analysis.

*Separation conditions for donepezil and rivastigmine in plasma and brain samples*

An analytical column (Waters Spherisorb S5 W (250 mm × 4.6 i.d.; 5 μm particle size)) with guard column (Waters Spherisorb S5 W (30 mm × 4.6 mm i.d.)) was used for the analysis. The mobile phase contained acetonitrile and 50 mM sodium dihydrogen phosphate (17:83; v/v). The pH was adjusted to 3.1 with phosphoric acid (13). The sample volume was 40 µL for plasma samples and 90 µL for brain-tissue samples. All chromatograms were obtained at a conditioned temperature (50 °C), and the flow rate was 1.3 mL/min (13). The described analytical method is sensitive for donepezil and also for rivastigmine, and may be used for both acetylcholinesterase inhibitors (both inhibitors were used as internal standards). The samples were analysed by UV detector, and the maximum wavelength of donepezil was 210 nm and 200 nm for rivastigmine. 

*Animal treatment*

Male adult Wistar rats (body weight 230 ± 30 g; Anlab Inc., Prague, Czech Republic) were used for pharmacokinetics study. The animals were kept under recommended conditions: standard laboratory food and tap water were available *ad libitum*. All experimental procedures and protocols were reviewed and approved by the Ethics Committee of the Faculty of Military Health Sciences, University of Defense, Hradec Kralove, Czech Republic. Donepezil and rivastigmine were freshly dissolved in saline solution (0.9% w/v NaCl) before each application (0.1 mL/100 g of animal weight). Doses were calculated according to recommended human dosages, and were delivered by i.m. injection: donepezil 143 µg/kg; rivastigmine 137 µg/kg. Blood samples were collected directly from the heart into heparinized 1.5 mL polythene tubes after 5, 15, 30, 45, 60, 120 and 240 min, and after 24 h (n = 6; six animals in each time interval). The samples were immediately centrifuged at 3000 ×g for 10 min (10 °C), and the plasma obtained was stored at −80 °C until analysis. 

Since the blood within the brain vessels also contained the tested compounds, it is not appropriate to measure the brain tissue levels of donepezil and rivastigmine directly from untreated brain homogenate. Hence, the animals were perfused transcardially with saline solution (0.9% NaCl) for 8 min (50 mL/min) (14). After perfusion, the skull was opened and the brain was checked visually and subsequently carefully removed; time intervals correspond with blood sampling (5, 15, 30, 45, 60, 120 and 240 min, and after 24 hours; n = 6; the same animals were used). All brains were stored at –80 °C until HPLC analysis (15).

Standard noncompartmental analysis was performed using Kinetica software, version 4.0 (InnaPhase Corporation, Thermo Fisher Scientific Inc., Waltham, MA, USA). Maximum concentration (C_max_) and the time to the maximum concentration (T_max_) were determined directly from the observed data. The area under the mean plasma concentration–time curve from zero up to infinity (AUC_total_) was determined as the sum of the AUC_0–24 h_ and of the extrapolated part, *i.e.* the ratio of the concentration predicted at the time interval of 24 h and the terminal rate constant λz. The λz was estimated using linear regression of the log-transformed concentrations at interval from 45 min to 24 h plotted against time. The half-life was calculated as t_1/2 _= ln(2)/λz. Other statistical analysis was performed using GraphPad Prism, version 5.0 (GraphPad Software, San Diego, California, USA).

*Sample preparation procedure*

One millilitre of plasma was spiked with IS working solution (20 µL of 10% stock solution). The extraction was performed with 20 µL of 1 M sodium hydroxide and 3 mL of 1-butanol/n-hexane (2:98; v/v). Subsequently, the samples were shaken intensely for 5 min and centrifuged at 11,000 g at 10 °C for 10 min. The entire organic layer was separated into a new tube, and re-extraction was performed with 100 µL of 0.1% acetic acid (13). The mixture was shaken intensely and centrifuged. The lower aqueous phase was separated and directly injected into the HPLC system.

Brain tissues (whole brains) were mechanically crushed under liquid nitrogen. 0.5 g of brain tissue was added to 1 mL 0.1% acetic acid and the mixture homogenized by ultrasound homogenizer UP50H (Hielscher - Ultrasound Technology, Germany) for 30 s, and subsequently centrifuged at 14,000 ×*g* at 10 °C for 15 min. Eight-hundred microliter of supernatant was subjected to liquid-liquid extraction as previously described. All assessments were performed in triplicate.

*Preparation of calibration standards and calculation of assayed concentrations*

A seven-point calibration curve based on the peak area ratio of donepezil or rivastigmine/IS showed a good linear relationship over the range of 0.1–100 ng/mL, with a regression coefficient r^2^ = 0.9953 - 0.9999. The LLOQ in rat plasma was 0.5 ng/mL for donepezil and 0.8 ng/mL for rivastigmine, and the LLOQ in rat brain was 1.0 ng/mL for donepezil and 1.1 ng/mL for rivastigmine. Both inter- and intra-day precision and accuracy for all the investigated concentrations of donepezil and rivastigmine in rat plasma/brain homogenate were within acceptable limits (Valis *et al.*, 2017). The samples were measured directly after the calibration. Regression analysis was performed by the method of least-squares using Prism 4 (Graph Pad Software, USA).

## Results

*Tolerability*

All animals tolerated the application of both drugs donepezil and rivastigmine without any signs of discomfort, such as gastrointestinal discomfort, salivation or lacrimation, or other symptoms typical for cholinesterase inhibitor intoxication, during the 240-minute follow-up period.

*Changes in plasma concentration and central nervous system targeting*

**Figure 2:**
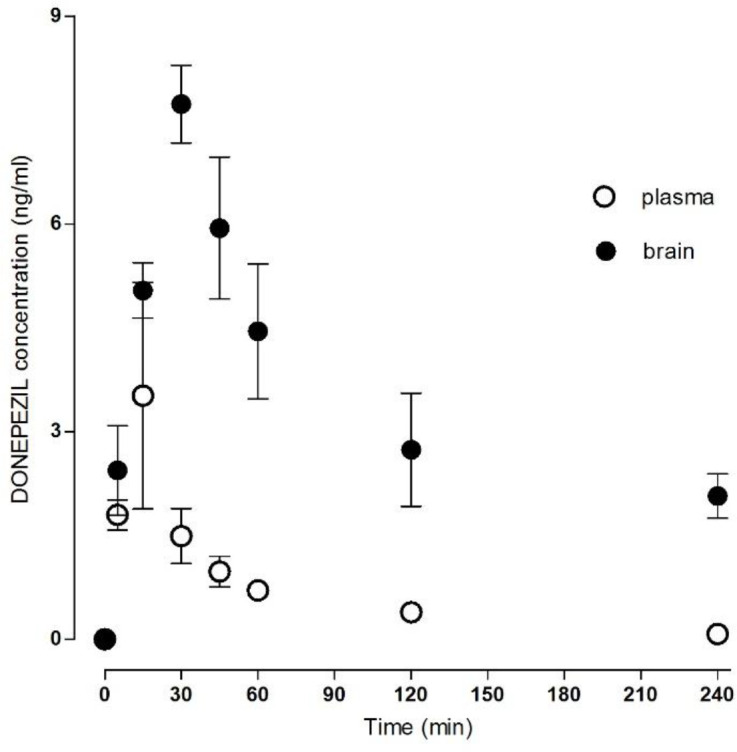
Plasma/brain concentration–time profile of donepezil after single intramuscular application of 143 µg/kg. Data are mean ± SD (n = 6)

**Figure 3:**
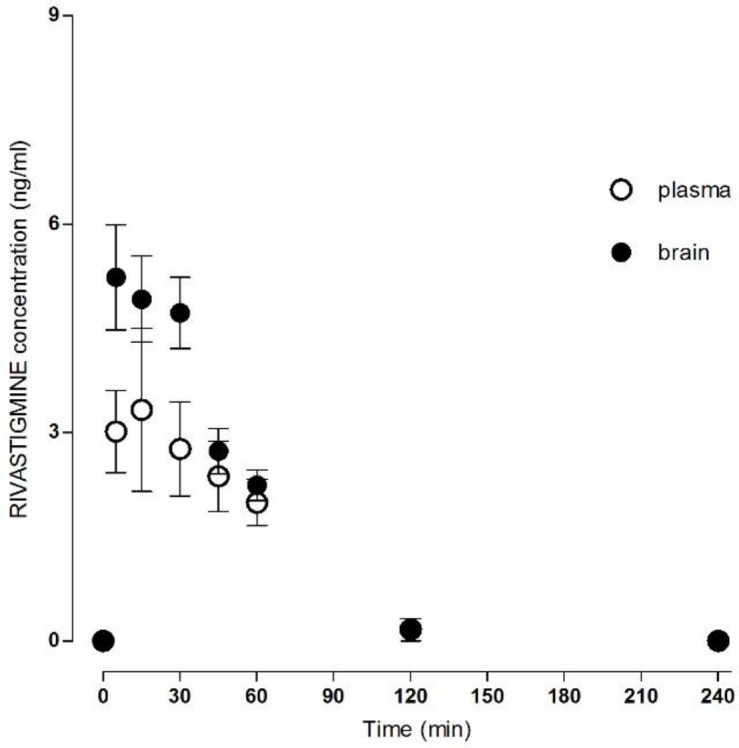
Plasma/brain concentration–time profile of rivastigmine after single intramuscular application of 137 µg/kg. Data are mean ± SD (n = 6).

The method was successfully applied to measure the donepezil and rivastigmine concentration in rat plasma and brain homogenate after i.m. administration (Table 1). The mean concentration–time donepezil plasma and brain profile is shown in Figure 2 and rivastigmine profile in Figure 3. 

The absorption after i.m. application was relatively fast; the C_max_ in plasma was 3.65 ± 1.42 ng/mL for donepezil and 4.96 ± 0.67 ng/mL for rivastigmine at T_max_ of 22.00 ± 6.26 min for donepezil and 25.00 ± 6.16 min for rivastigmine. The AUC_total_ was 156.53 ± 23.36 min.ng/mL for donepezil and 201.85 ± 8.99 min.ng/mL for rivastigmine.

The C_max_ in the brain tissue was 8.34 ± 0.34 ng/mL for donepezil and 6.18 ± 0.40 ng/mL for rivastigmine, and the T_max_ 36.00 ± 3.29 min for donepezil and 17.00 ± 5.02 min for rivastigmine. The AUC_total_ was 1389.67 ± 159.65 min.ng/mL for donepezil and 350.65 ± 33.64 min.ng/mL for rivastigmine. All calculated plasma and brain pharmacokinetics parameters are summarized in Table 2.

**Table 1:** Time-concentration changes of donepezil (143 µg/kg) and rivastigmine (137 µg/kg) in rat plasma and brain after single intramuscular application.

**Time** **(min)**	**Donepezil concentration (ng/mL)**			**Rivastigmine concentration (ng/mL)**		
	**Plasma** **mean ± SEM**	**Brain** **mean ± SEM**	**Ratio plasma:brain** **(%)**	**Plasma** **mean ± SEM**	**Brain** **mean ± SEM**	**Ratio plasma:brain** **(%)**
0	0.00 ± 0.00	0.00 ± 0.00	0.00	0.00 ± 0.00	0.00 ± 0.00	0.00
5	1.80 ± 0.22	2.44 ± 0.65	135.56	3.01 ± 0.59	5.23 ± 0.76	173.75
15	3.52 ± 1.63	5.05 ± 0.40	143.47	3.33 ± 1.17	4.92 ± 0.62	147.74
30	1.49 ± 0.40	7.73 ± 0.56	518.79	2.77 ± 0.68	4.72 ± 0.51	170.39
45	0.98 ± 0.22	5.94 ± 1.02	606.12	2.37 ± 0.51	2.73 ± 0.32	115.19
60	0.71 ± 0.05	4.45 ± 0.97	626.76	1.99 ± 0.33	2.24 ± 0.22	112.56
120	0.39 ± 0.10	3.42 ± 0.58	876.92	0.17 ± 0.15	0.03 ± 0.02	17.65
240	0.08 ± 0.03	2.07 ± 0.32	2587.5	0.00 ± 0.00	0.00 ± 0.00	0.00
24 h	0.00 ± 0.00	0.00 ± 0.00	0.00	0.00 ± 0.00	0.00 ± 0.00	0.00

**Table 2:** Pharmacokinetic parameters obtained after a single i.m. injection of donepezil (143 µg/kg) and rivastigmine (137 µg/kg)

**Plasma**	**Donepezil**	**Rivastigmine**
Cmax (ng/mL)	3.65 ± 1.42	4.96 ± 0.67
Tmax (min)	22.00 ± 6.26	25.00 ± 6.16
AUCtotal (min.ng/mL)	156.53 ± 23.36	201.85 ± 8.99
λz (1/min)	0.0141 ± 0.0023	0.0401 ± 0.0061
Half - life (min)	57.06 ± 10.10	19.82 ± 3.42
MRT (min)	75.58 ± 15.48	43.38 ± 3.52
CL (mL/min/kg)/F	1.00 ± 0.13	0.69 ± 0.03
Vz (l/kg)/F	82.06 ± 13.91	19.81 ± 3.87
**Brain**		
Cmax (ng/mL)	8.34 ± 0.34	6.18 ± 0.40
Tmax (min)	36.00 ± 3.29	17.00 ± 5.02
AUCtotal (min.ng/mL)	1389.67 ± 159.65	350.65 ± 33.64
λz (1/min)	0.0056 ± 0.0006	0.0251 ± 0.0058
Half - life (min)	131.13 ± 14.53	38.26 ± 10.69

All values are mean ± SEM. (n = 6). C_max_ = max. donepezil and rivastigmine plasma concentration, T_max_ = time of C_max_, AUC_total _= area under the concentration-time curve from zero up to infinity/bioavailability, λz = terminal rate constant, half-life in elimination phase, MRT = mean residence time of molecule in the body, CL = clearance and Vz = volume of distribution during terminal phase.

## Discussion

Donepezil (selective AChE inhibitor) and rivastigmine (pseudo-irreversible AChE and BChE inhibitor) belong to the small regimen of cholinesterase inhibitors that are used clinically as palliative AD treatment. The most significant therapeutic effect is stabilization of cognitive function, but in only approximately 50% of patients for a period of 1 year, and approximately 20% for more than 2 years (16). Although the continued application of cholinesterase inhibitors leads to progressive loss of efficacy, alternative strategies for improvement of AD therapy are currently limited. 

Although pharmacokinetics data on rivastigmine, especially in terms of brain targeting, are still rare, there are existing preclinical and clinical data on donepezil. Donepezil plasma levels reach a steady state within three months and remain unchanged (17). According to some studies the plasma concentration of donepezil is dose-proportional: 30 ng/mL (5 mg/day; p.o.) and 60 ng/mL (10 mg/day; p.o.); cerebrospinal fluid (CSF) levels have been estimated as proportionately ten-times lower (18-20). Based on human acetylcholinesterase inhibition the human CSF donepezil fluctuation was assessed about 50% between the doses (19).

Clinical studies with direct assessment of CSF donepezil levels are rare: only one HPLC study exists. The plasma concentrations correspond to approximately 40 ng/mL 12 h after application, and 30 ng/mL after 24 h; CSF concentrations in response to treatment with 10 mg/day p.o. were 5.2 ng/mL 12 h after application and 7.5 ng/mL after 24 h (7). Unfortunately, the available data do not allow calculation of advanced pharmacokinetics parameters such as C_max_, T_max_, AUCs *etc.*

Based on clinical study results, the CSF donepezil levels are lower than plasma donepezil levels: ten-times (acetylcholinesterase inhibition) or four/eight-times depending on time interval (HPLC study) (7, 19). However, brain-tissue donepezil levels are greater than plasma donepezil levels. The brain donepezil levels exceed plasma levels relatively quickly, and stay significantly higher at all time intervals (Table 1; plasma: brain ratios). Similar results were published by Geerts *et al.* (21) where the plasma AUC_1-6 h_ was significantly lower than brain AUC_1-6 h_ in rats after s.c. application. The AUC plasma/brain ratios varied considerably, different from 1.66 after a 0.05 mg/kg dose to 32.12 after a 1.5 mg/kg dose in rats. Brain accumulation of donepezil was also found in mice and rabbits (21). Similar clinical studies assessing the distribution of donepezil into selected human brain segments are not available. Although some preclinical and clinical data on donepezil have been published, its real pharmacokinetics and pharmacodynamic effect in human brain is still only estimated.

Preclinical and clinical data about rivastigmine are rare and are limited to acetylcholinesterase/butyrylcholinesterase inhibition studies. This is the first report directly comparing donepezil and rivastigmine plasma and brain levels in *in-vivo* study. Plasma levels of rivastigmine increased transiently with a calculated maximum of 4.96 ± 0.67 ng/mL at 25.00 ± 6.16 min after i.m. application. The rivastigmine plasma levels were significantly lower than the brain tissue levels. The maximum rivastigmine brain level was calculated as 6.18 ± 0.40 ng/mL at 17.00 ± 5.02 min. Brain clearance was faster for rivastigmine than for donepezil, and the plasma: brain ratios are not as significant as for donepezil. This is in line with estimated clinical data (22, 23). The result suggests that there is no accumulation of rivastigmine over the longer term in rats. In human AD treatment the donepezil is applied once and rivastigmine twice per day. Based on the aforementioned clinical, preclinical and experimental data, donepezil is frequently used in the synthesis of novel hybrid molecules, with variable potential therapeutic anti-AD effects (24-26). On the other hand, the application routine of rivastigmine has been simplified by introduction of patch/transdermal system with better tolerability for patients (27).

Based on all previously published preclinical and clinical data, it can be expected that estimation of the human CSF/brain pharmacokinetics profile would be complex. The majority of preclinical studies, including our study, are performed on young laboratory animals. The effect of age on the drug pharmacokinetics profile can be estimated (28); distribution into the CNS can be also influenced by body fat/water composition and decreased transporter activity (*e.g.* P-glycoprotein), and so the permeability of the blood-brain barrier could alter with ageing (29-31). Some data have been published concerning the differences in donepezil pharmacokinetics profile between young and old animals (31). The donepezil brain concentration was significantly higher (1.8-fold) in old rats compared to young animals. 

The trend for increased donepezil brain concentrations observed in older rats makes it difficult to find rules for clinical pharmacokinetics profiling and estimation of the real pharmacodynamic response. These differences and difficulties can be expected also in the case of application of the other drugs commonly used in AD treatment strategy. 

## Conclusion

Pharmacokinetics studies already exist, describing brain-targeting of donepezil, but comparable/similar information about rivastigmine is still rare. Preclinical and clinical data for rivastigmine are limited to cholinesterase inhibition. Pharmacokinetics data based on pharmacodynamic interaction may not be accurate. According to our knowledge, this is the first report directly comparing the brain levels of donepezil and rivastigmine. 

Both compounds showed ability to target the central nervous system, with brain concentrations exceeding those in plasma. Maximum brain concentration after i.m. administration was reached in the 36th (8.34 ± 0.34 ng/mL) and 17^th^ min (6.18 ± 0.40 ng/mL) respectively for donepezil and rivastigmine. The differences in brain profile can be most easily expressed by plasma/brain AUCtotal ratios: donepezil ratio in the brain was nine-times higher than in plasma and rivastigmine ratio was less than two-times higher than in plasma.
